# The Effect of Peri-Implant Therapy on the Expression of Th17-Related Cytokines in Patients with Peri-Implant Mucositis and Peri-Implantitis: A Prospective Longitudinal Study

**DOI:** 10.3390/jcm14020340

**Published:** 2025-01-08

**Authors:** Líssya Tomaz da Costa Gonçalves, Glaucia Schuindt Teixeira Neves, Alexandre Marques Paes da Silva, Daniel de Moraes Telles, Carlos Marcelo da Silva Figueredo, Eduardo José Veras Lourenço, Mayla Kezy Silva Teixeira

**Affiliations:** 1 Department of Prosthodontics, School of Dentistry, Rio de Janeiro State University, Rio de Janeiro 20551-030, Brazil; lissyagoncalves@gmail.com (L.T.d.C.G.); glauciasteixeira@hotmail.com (G.S.T.N.); xandemps@gmail.com (A.M.P.d.S.); dmtelles@uerj.br (D.d.M.T.); everaslourenco@outlook.com (E.J.V.L.); maylakezy@hotmail.com (M.K.S.T.); 2 School of Medicine and Dentistry, Griffith University, Queensland 4222, Australia; 3 Division of Oral Diseases, Department of Dental Medicine, Karolinska Institute, 171 77 Stockholm, Sweden

**Keywords:** dental implants, peri-implantitis, mucositis, therapy, cytokines, Th17 cells

## Abstract

**Background/Objectives**: Cytokines related to the Th17 response have been associated with peri-implant diseases; however, the effect of peri-implant therapy on their modulation remains underexplored. To evaluate the effect of peri-implant therapy on the expression of cytokines related to the Th17 response in the peri-implant crevicular fluid (PICF) (GM-CSF, IFN-γ, IL-1β, IL-4, IL-6, IL-10, IL-12 (p70), IL-17A, IL-21, IL-23, and TNF-α) of partially edentulous patients with peri-implant disease (PID). **Methods**: Thirty-seven systemically healthy individuals presenting with peri-implant mucositis (PIM) (n = 20) or peri-implantitis (PI) (n = 17) were treated and evaluated at baseline (T0) and three months after therapy (T1). Clinical parameters (probing depth (PD), clinical attachment level (CAL), plaque index, and bleeding on probing index (BoP), were evaluated. The PIM group underwent non-surgical therapy, while the PI group received a surgical approach. PICF was collected with absorbent paper strips and analyzed with a multiplex assay. **Results**: Eighty-eight implants were treated in 37 patients (56 in the PIM group and 32 in the PI group). After therapy, significant reductions in PD, CAL, plaque index, and BoP were observed in the PIM group (*p* < 0.05). In the PI group, significant reductions in PD, CAL, and BoP were noted (*p* < 0.05). The PIM group showed a significant reduction of IL-17A and TNF-α after therapy, while the PI group showed a significant reduction of IL-1β, IL-6, and TNF-α (*p* < 0.05). **Conclusions**: The peri-implant therapy for patients with PID reduced the expression of cytokines related to the Th17 response in PICF.

## 1. Introduction

Peri-implant mucositis (PIM) is an inflammatory condition characterized by inflammation of peri-implant tissues with bleeding and/or suppuration on probing, without accompanying bone loss. Peri-implantitis (PI) is distinguished by progressive bone loss, which may result in implant loss [1,2]. The prevalence of peri-implant diseases (PID) averages 47% for PIM and 20% for PI [3]. As the number of patients undergoing dental implant rehabilitation increases, biological complications also rise, presenting a relevant concern in dentistry [4,5,6,7].

The primary cause of inflammation development is the presence of biofilm in peri-implant tissues, which stimulates an immune host response [1]. The release of pro-inflammatory cytokines plays a crucial role in influencing the onset and progression of PID [7,8]. The precise significance of cytokines in PID remains still not clear, but it is recognized that a complex network of molecular interactions is associated with peri-implant inflammation and bone resorption in response to bacterial factors [9]. The inflammatory response leads to an increase in peri-implant crevicular fluid (PICF) production, enabling the identification of biomarkers through its analysis [10,11]. PICF collection is a simple, reproducible, and non-invasive technique that allows the assessment of specific sites of interest, acting as an important tool for monitoring disease activity [12,13,14].

T helper cells encompass seven subpopulations (Th1, Th2, Th9, Th17, Th22, Th, Tfh, Treg) and are pivotal in orchestrating the host immune response against bacterial aggression [15]. Th17 population is particularly significant in autoimmune and allergic diseases, as well as in host defense against pathogens [16,17]. In this context, understanding the activity of these cells is important to elucidate the immunoinflammatory mechanisms underlying peri-implant tissue destruction [18]. Previous cross-sectional studies have investigated the presence of cytokines from this group in PICF of patients with PIM and PI, establishing an association with disease activity [11,19]. However, the expression of these cytokines seems not to be different in PI and PIM, despite the presence of osteoclastogenesis [19]. As far as we know, prospective studies evaluating the expression of cytokines related to Th17 response and the effects of peri-implant therapy on modulating these biomarkers remain lacking.

Achieving success in peri-implant treatment poses a considerable challenge [20,21]. For patients with PIM, the approach usually involves non-surgical methods aimed at removing biofilm and calculus and providing oral hygiene instructions [22]. However, in cases of PI, non-surgical therapy seems ineffective in reducing probing depth (PD) and bleeding on probing [21,23,24]. As a result, surgical therapy becomes frequently necessary to improve access to the infected site and ensure satisfactory cleaning of the implant, which is crucial to obtaining favorable outcomes in PI treatment [25].

Therefore, this study aimed to evaluate the effect of peri-implant therapy on the expression of cytokines related to Th17 response (GM-CSF, IFN-γ, IL-1β, IL-4, IL-6, IL-10, IL-12 (p70), IL-17A, IL-21, IL-23, and TNF-α) in the peri-implant crevicular fluid of patients with peri-implant mucositis and peri-implantitis after a three-month follow-up.

## 2. Materials and Methods

### 2.1. Participants and Study Setting

This study was conducted at the School of Dentistry, State University of Rio de Janeiro, and obtained approval from the Research Ethics Committee under the number 69156722.5.0000.5259. All research participants were duly informed and signed a free and informed consent form, following the Declaration of Helsinki.

The individuals sampled for this present study were chosen based on the following inclusion criteria [26]:

- Being systemically healthy or having controlled systemic conditions;
- Being partially edentulous, with at least two osseointegrated implants affected by peri-implant disease (PID);
- Having implant prosthetics that have been in function for a minimum of six months.

Exclusion criteria included individuals who [26]:

- Had received periodontal or peri-implant treatment within six months before the study commencement;
- Were pregnant or breastfeeding;
- Were smokers;
- Had taken antibiotics and anti-inflammatories within the last three months;
- Had taken antiresorptive drugs within the last two years;
- Had undergone radiotherapy, chemotherapy, or iodine therapy within the last two years.

The individuals were allocated in groups according to the following criteria:

Peri-implant mucositis group (PIM): clinical signs of inflammation, bleeding, and/or suppuration on probing; absence of radiographic bone loss beyond initial levels of bone remodeling [1,27].

Peri-implantitis group (PI): clinical signs of inflammation, bleeding, and/or suppuration on probing, accompanied by radiographic bone loss beyond initial levels of bone remodeling, compared to previous radiographs. In the absence of previous exams, criteria included probing depth ≥ 6 mm and radiographic bone loss ≥ 3 mm [1,27].

### 2.2. Clinical Examination

Each participant underwent both a clinical examination and a complete periapical full-mouth X-ray. The clinical examination was further divided into anamnesis and physical examination. During the anamnesis, demographic data and information regarding the time since their last periodontal and peri-implant maintenance therapy (PIMT) were gathered. The intraoral examination involved a complete periodontal chart, performed by a previously calibrated operator.

A standardized millimeter periodontal probe was used, and the measurements were rounded to the nearest millimeter (Hu-Friedy^®^ PCP15, Chicago, IL, USA). Probing was performed at six sites per tooth/implant, assessing parameters such as PD (mm), clinical attachment level (CAL) (mm), bleeding on probing index (0 or 1), and visible plaque index (0 or 1) [28]. All clinical parameters and radiographs were evaluated before (baseline) and after three months of therapy (T1).

### 2.3. Peri-Implant Crevicular Fluid Collection

PICF collection was conducted under relative isolation, with cotton rolls around the implants, and surfaces were gently dried to prevent contamination from plaque or saliva. Two to three sites were selected per group, based on the deepest probing depth. Standardized absorbent paper strips (Periopaper^®^–Oraflow, Smithtown, NY, USA) were inserted 1–2 mm into the peri-implant sulcus, without traumatizing the tissues, remaining for up to 30 s [29]. Any samples contaminated with blood were discarded, while strips containing the fluid were stored in the same Eppendorf-type microtube, containing 200 μL of PBS buffer solution and 10 μL of protease inhibitor (Sigma-Aldrich, St. Louis, MO, USA). After 45 min, the paper strips were discarded, and the solution was centrifuged at 8000 rpm for five minutes in a laboratory centrifuge (NT800-Novatécnica, Piracicaba, SP, Brazil). Subsequently, the sample was transferred to a threaded microtube and frozen at −70 °C until analysis. FCPI collection was performed at baseline and T1.

### 2.4. Peri-Implant Therapy

The treatment of peri-implant diseases adhered to established protocols from prior consensus studies [30,31].

For the PIM group, a non-surgical approach was adopted, which included oral hygiene instruction, plaque control, and scaling of the implants using non-metallic manual curettes (Implacare-Hu-Friedy^®^, Chicago, IL, USA). Additionally, polishing procedures were conducted with a rubber cup, employing prophylactic paste (Maquira Shine-Maquira, Maringá, PR, Brazil) and bicarbonate jet (Jetlaxis Uno-Schuster, Santa Maria, RS, Brazil).

For the PI group, the same non-surgical approach was applied, followed by a surgical intervention performed by a single experienced periodontist after one month. If the prosthesis was screwed-retained, it was removed before surgery and subsequently reinstalled after the procedure. Surgery was performed using a total flap, with intrasulcular incisions made extending to one tooth or implant adjacent to each side. Granulation tissue was carefully removed with a Gracey curette (Hu-Friedy^®^, Chicago, IL, USA). The area was irrigated with sterile saline, and biofilm and calculus removal were performed using hand instruments. Polishing was performed with a bicarbonate jet (Jetlaxis Uno-Schuster, Santa Maria, RS, Brazil), a Robinson brush, and prophylactic paste (Maquira Shine-Maquira, Maringá, PR, Brazil). The region was then irrigated with 2% chlorhexidine solution (Chlorhexidine 2%-Maquira, Maringá, PR, Brazil), and the flap was repositioned and sutured. Postoperative medication (Amoxicillin 500 mg-8 in 8 h, for 7 days; Nimesulide 100 mg-12 in 12 h, for 3 days and Dipyrone-6 in 6 h, for 2 days) and mouthwash (chlorhexidine digluconate 0.12%-twice a day, for 14 days) were prescribed, with instructions not to brush the region until sutures were removed. The suture was removed 14 days after the surgical procedure.

### 2.5. Multiplex Assay

Cytokine levels (GM-CSF, IFN-γ, IL-1β, IL-4, IL-6, IL-10, IL-12 (p70), IL-17A, IL-21, IL-23, and TNF-α) were assessed through a multiplex microsphere immunoassay (Bioplex^®^ 200-Bio-Rad, Hercules, CA, USA). Twenty-five microliters of each fluid sample were analyzed using a commercially available custom kit-Milliplex^®^ Human High Sensitivity T Cell Magnetic Bead Panel Kit (Merck Millipore, Burlington, MA, USA), following the manufacturer’s instructions, using a 96-well plate.

**Figure 1:**
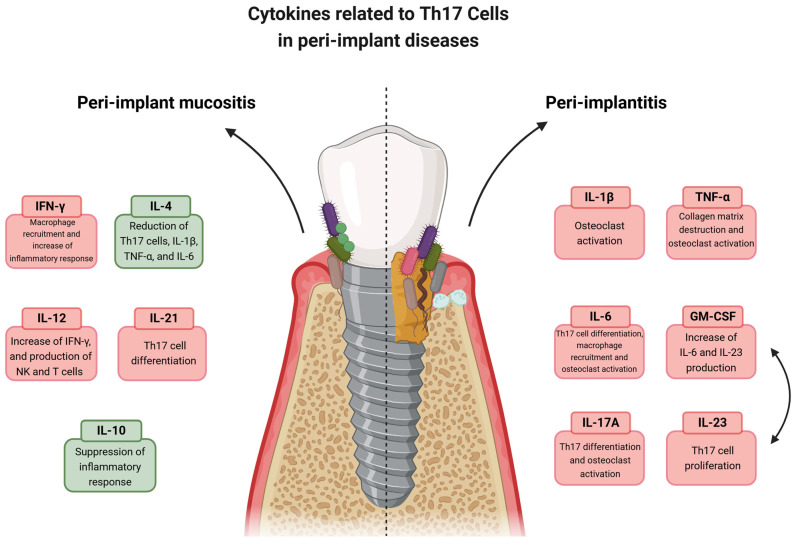
Illustration of the functions of cytokines related to Th17 cells in peri-implant diseases. The left side represents peri-implant mucositis, characterized by biofilm-induced inflammation without bone loss. The right side illustrates peri-implantitis, with biofilm-induced inflammation, bone loss, and the presence of osteoclasts. The cytokines evaluated in this study are highlighted in the illustration with their functions in the inflammatory response. A bidirectional arrow between GM-CSF and IL-23 indicates the positive feedback loop between these cytokines.

Figure 1 provides a summary of the functions of the assessed cytokines in peri-implant diseases.

### 2.6. Statistical Analyses

Statistical analyses were conducted using SPSS, version 24 (IBM Corporation, Armonk, NY, USA). Data normality was assessed using the Kolmogorov-Smirnov test. Continuous variables were presented as mean and standard deviation, while categorical variables were presented as frequencies. The Mann-Whitney U test for independent samples was employed to compare continuous variables between groups, while the Chi-square test was utilized to compare frequencies between groups. Within-group analyses at different time points were conducted using the Wilcoxon test, adopting a significance level of *p* < 0.05. Pearson’s correlation coefficient was used to assess the correlation between cytokine levels, while Spearman’s correlation coefficient was used to assess the relationship between cytokine levels and clinical results. Significance levels for correlation were set at both *p* < 0.05 and *p* < 0.01.

## 3. Results

### 3.1. Demographic Data

The study included 37 participants who were either systemically healthy or had controlled systemic conditions. The demographic data are presented in Table 1.

### 3.2. Clinical Results

In the analyses of the PIM group, the treated implants showed a significant reduction of PD, CAL, and percentage of PId and BoP values (*p* = 0.001, *p* = < 0.001, *p* = 0.003, and *p* = < 0.001, respectively). The outcomes are presented in Table 2.

In the analyses of the PI group, the treated implants showed a reduction statistically significant of PD, CAL, and % BoP values (*p* = < 0.001, *p* = 0.004, and *p* = < 0.001, respectively). The outcomes are presented in Table 3.

### 3.3. Description of Implants

Eighty-eight implants were evaluated: 56 in the PIM group (63.63%) and 32 in the PI group (36.37%). The average functional time of the implants was 88.05 months (±59.53). It was evaluated categorically, divided into less than five years, between five and ten years, and more than ten years. No statistically significant differences were observed among these categories (*p* = 0.338).

The implants were evaluated according to their location in the arch, type of prosthetic platform, prosthetic connection (cemented or screwed), and whether they were splinted or non-splinted prostheses. The distribution of implants with PIM in the upper arch was significantly greater than those with PI (*p* = 0.002). In both groups, the prevalence of inflamed sites was significantly higher in the posterior region of the arches (*p* = < 0.001). Additionally, the prevalence of PIM was significantly higher in Morse Taper (MT) platform implants (*p* = < 0.001), while implants with PI had a significantly higher prevalence in the External Hexagon (EH) platform. Complete data are described in Table 4.

### 3.4. Immunological Results

In the analysis between the PIM and PI groups, no statistically significant differences were observed at baseline and T1 (*p* > 0.05). The cytokine levels were presented in total quantity (pg).

In the PIM group, a statistically significant reduction in the expression of IL-17A (*p* = 0.010) and TNF-α (*p* = 0.035) was observed after therapy.

In the PI group, there was a statistically significant reduction in the expression of IL-1β (*p* = 0.049), IL-6 (*p* = 0.01), and TNF-α (*p* = 0.011), with a trend towards a reduction in IFN-γ (*p* = 0.059).

**Figure 2:**
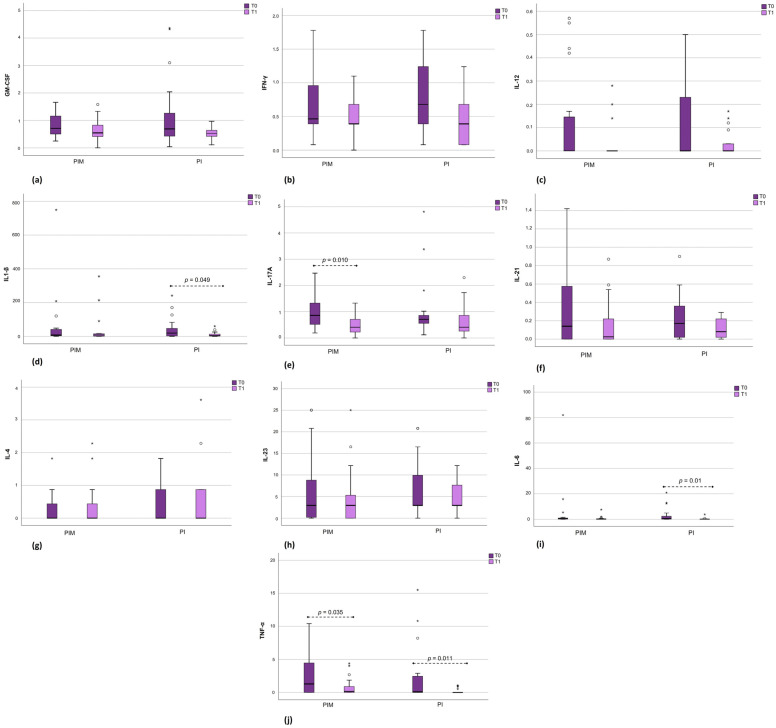
(**a**–**j**) Representation of biomarker expression results before and after therapy. PIM—Peri-implant mucositis; PI—Peri-implantitis. Results are presented in boxplots, with the left side referring to the PIM group (before and after therapy) and the right side referring to the PI group (before and after therapy). The * and circles represent outliers: * indicates values two times the standard deviation (SD), while circles indicate values three times the SD. When the *p*-value was < 0.05, the values were included in the figure. The boxplot graphs were created with the SPSS program (version 24).

The immunological data are described in Figure 2. IL-10 levels were below the detection limit and, therefore, were not included in the presentation.

### 3.5. Correlation Results

In the PIM group, after therapy, a significant negative correlation was observed between IFN-γ, IL-12, and IL-23 with PD. Additionally, IL-12 showed a significant negative correlation with the percentage of bleeding.

In the PI group, before therapy, there was a significant positive correlation between the levels of IFN-γ and IL-21 with PD. After therapy, IFN-γ showed a significant negative correlation with PD, while IL-21 showed a significant positive correlation with the percentage of plaque.

**Figure 3:**
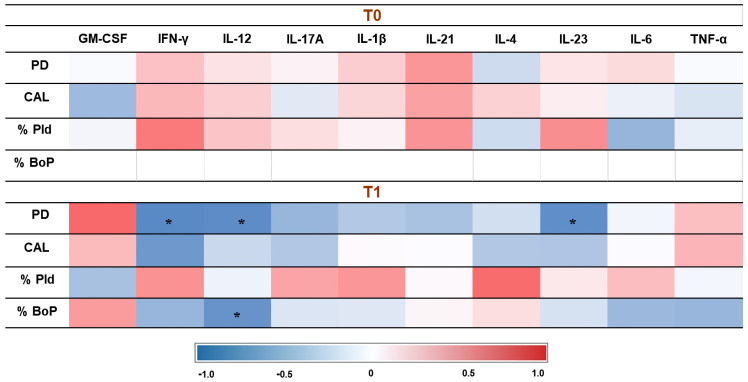
Correlation between cytokines and clinical data in PIM group. PD: probing depth; CAL: clinical attachment level; % PID: plaque index; %BoP: bleeding on probing. The correlation coefficient was obtained by Spearman’s correlation test. *: *p* < 0.05 level.

**Figure 4:**
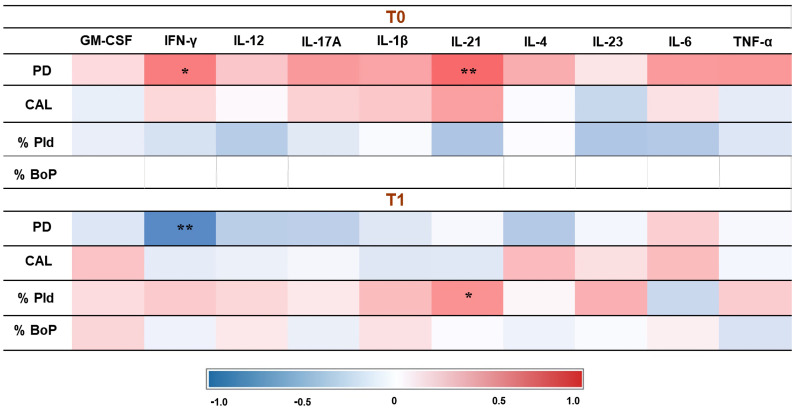
Correlation between cytokines and clinical data in the PI group. PD: probing depth; CAL: clinical attachment level; % PID: plaque index; %BoP: bleeding on probing. The correlation coefficient obtained by Spearman’s correlation test. **: *p* < 0.01; *: *p* < 0.05 level.

The correlations between cytokines and clinical data at both times (baseline and T1) in the PIM group are represented in Figure 3, whereas the correlations between cytokines and clinical data in the PI group are represented in Figure 4.

**Figure 5:**
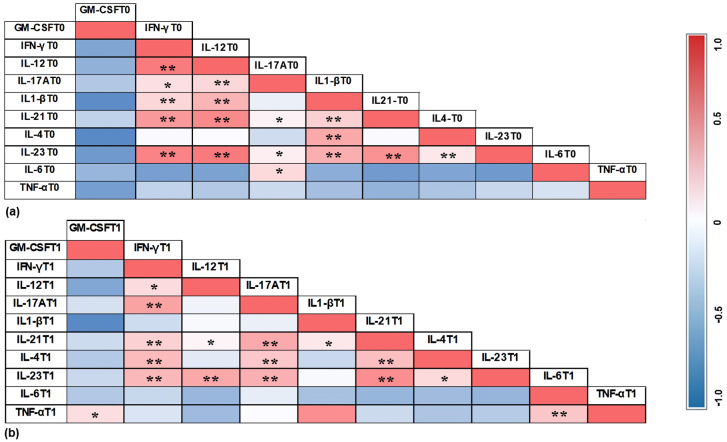
Correlation between cytokines in the PIM group in T0 (**a**) and T1 (**b**). The correlation coefficient was obtained by Pearson’s correlation test. **: *p* < 0.01; *: *p* < 0.05.

**Figure 6:**
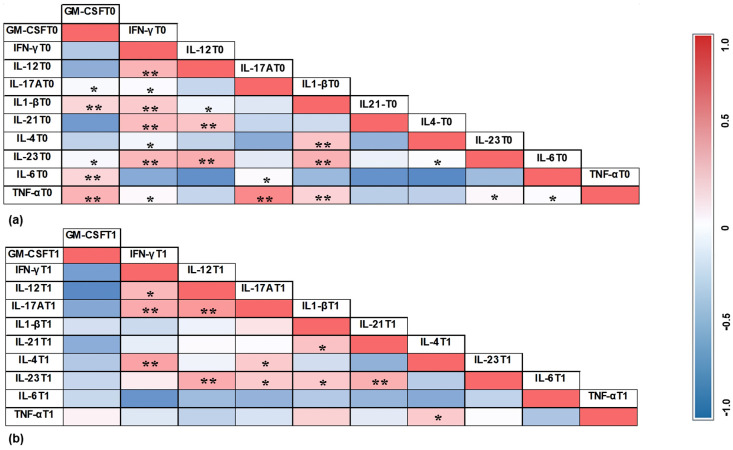
Correlation between cytokines in PI group in T0 (**a**) and T1 (**b**). The correlation coefficient was obtained by Pearson’s correlation test. **: *p* < 0.01; *: *p* < 0.05.

The correlations between cytokines in the PIM group in T0 and T1 are illustrated in Figure 5, while the correlations between cytokines in the PI group in T0 and T1 are demonstrated in Figure 6.

## 4. Discussion

In the present study, peri-implant therapy resulted in a reduction of pro-inflammatory biomarker expression in PICF from both the PIM and PI groups. Specifically, the PIM group showed a significant decrease in IL17-A and TNF-α, while the PI group exhibited a significant reduction in IL1-β, IL-6, and TNF-α. Additionally, there was a decreased tendency towards IFN-γ levels in the PI group, suggesting that the treatment may modulate this cytokine expression. Health implants were not included in the analysis, as the volume of PICF depends on the level of inflammation and PD [32], which probably would present a lower quantity in non-inflamed sites.

The observed significant reduction in IL-17A levels in patients with PIM after therapy suggests a positive modulation of the Th17 response, as this cytokine is involved in the differentiation of Th17 cells, osteoclast activation, and the recruitment of defense cells and pro-inflammatory cytokines such as IL1-β, IL-6, IL-8, G-CSF, GM-CSF, and TNF-α [33,34,35]. To the best of our knowledge, only one longitudinal study has assessed this cytokine in patients with PIM after therapy [36], also finding a significant reduction level. No prior studies have evaluated the effect of treatment on the modulation of IL-17A in PI. However, in our study, the reduction of IL-17A in the PI group did not reach statistical significance.

This study demonstrated a significant reduction in TNF-α levels in both the PIM and PI groups and a decrease in IL-1β levels specifically in the PI group after three months of therapy. These findings align with previous research, which also reported a significant reduction in TNF-α levels in the PICF of PIM patients following therapy [37]. Similarly, significant reductions in both TNF-α and IL-1β levels in the PICF of PI patients have been observed [38,39,40,41]. High levels of these biomarkers are typically found in the PICF of patients with PID [13,42,43,44,45,46], and they are considered predictive of disease progression [7]. Furthermore, our study revealed a significant positive correlation between TNF-α and IL-1β levels at baseline in the PI group, suggesting their coordinated activity in more inflamed sites. This positive correlation was not observed after therapy. The observed reduction in these biomarkers indicates a potential decrease in osteoclastic activity and inflammatory response [38,47].

The significant reduction in IL-6 levels in PI patients after therapy highlights the therapy’s positive impact on cytokine modulation. IL-6 is crucial in recruiting leukocytes, activating osteoclasts, and producing acute-phase proteins [48]. Consistent with our findings, previous studies have also reported decreased IL-6 levels in the PICF of PIM patients [37] and PI patients after therapy [41]. However, another study did not observe a statistically significant reduction in IL-6 levels six months after surgical therapy of PI, likely due to the initially low baseline levels of cytokine detection [49].

The tendency towards a significant reduction in IFN-γ levels in the PI group after therapy may suggest a potential association between IFN-γ and increased inflammation in PI. Our study also found a significant positive correlation between PD and IFN-γ at baseline in the PI group, with a negative correlation observed after therapy. This may indicate its role in increasing PD. This cytokine is known to promptly recruit macrophages, intensifying the inflammatory response [50] and accelerating periodontal disease progression [51]. However, it is important to note that the role of IFN-γ in the context of PID has been scarcely studied.

When comparing the PIM and PI groups at baseline, no statistically significant differences were found in the expression of any cytokine. This result suggests that once the inflammatory process begins, the expression of biomarkers remains similar regardless of the presence of bone loss. This finding is consistent with previous cross-sectional studies on Th17 response biomarker expression in the PICF of partially edentulous patients with PIM and PI [11,19].

IL-10 is a non-inflammatory cytokine that helps to suppress the inflammatory response and to protect the host [52,53]. In PIM and PI groups, IL-10 expression levels were below the detection limit, which can be attributed to the high pro-inflammatory activity present in both groups. The literature presents varied results: higher IL-10 expression in healthy patients compared to those with PI [38], higher IL-10 expression in the PI group compared to healthy controls [54], and no differences between groups [33,55].

The heat maps presented in Figure 4 and Figure 5 illustrate the correlations between cytokines in the two groups at different time points. Interestingly, in the PIM group, IL-17A showed a strong correlation with IL-6 before treatment, but this pattern was not maintained after therapy. Additionally, TNF-α in the PIM group did not show a significant positive correlation with any other cytokine. In contrast, in the PI group, TNF-α exhibited a significant positive correlation with GM-CSF, IL-17A, and IL-1β, which was no longer observed after treatment, suggesting a coordinated action of these cytokines during bone loss. Moreover, the correlation between GM-CSF and other cytokines was not observed in the PIM group but was identified in the PI group, with IL-17A, IL-1β, IL-6, and TNF-α. GM-CSF has the function of recruiting monocytes and dendritic cells in addition to promoting the increase of IL-6 and IL-23, which are involved in the differentiation of Th17 cells. The production of IL-23 enhances GM-CSF secretion, generating a positive feedback loop [56,57]. After treatment, these correlations were not observed, and the heat map reveals a negative tendency in the results. This finding may suggest the relevance of GM-CSF in the bone loss activity in peri-implant disease.

Our study found a higher prevalence of patients with PI when periodontal and peri-implant maintenance therapy (PIMT) was performed for more than one year. This result is consistent with findings from other studies [58,59,60,61,62] and highlights the importance of PIMT in preventing PI. The frequency of maintenance appointments must be established individually for each patient, based on their risk factors. However, a frequency of at least twice a year seems ideal for preventing PI [63,64]. Additionally, there was a notable trend of increased PI prevalence among patients with concomitant periodontitis at stage III. This condition is one of the factors most strongly associated with an increased risk of developing PI [4,59,65]. Although our findings were not statistically significant, this result may be attributed to the limited number of patients with periodontitis in the study.

This study observed a higher prevalence of PID in implants located in the posterior regions of the dental arches. This may be attributed to the greater difficulty in patients’ biofilm control of these areas, consistent with findings from previous studies [66,67,68,69,70]. However, there is no consensus on this issue, as some studies observed a higher prevalence of PI in implants located in the anterior region [69,70]. Additionally, there was a higher prevalence of PI in splinted prostheses, which can be explained by the increased complexity of biofilm removal in this type of prosthesis, making it more challenging for patients.

HE platform implants showed a higher prevalence of PI compared to CM platform implants. This difference can be attributed to the several advantages of CM over HE platforms, such as the internalization of the microgap between the abutment and implant, minimized micromovements in occlusal forces distribution, lower initial bone loss, improved bacterial sealing, and greater resistance to torque loss [71,72,73,74,75].

After therapy, there was a significant reduction in PD and the percentage of bleeding in the treated implants of the PIM and PI groups. However, most of the sites still exhibited bleeding, which may be related to the high plaque index observed. In the PI group, the plaque index was not significantly reduced, and in the PIM group, although it showed a significant reduction, it remained present in almost half of the evaluated implants. The persistence of bleeding after treatment has also been reported in previous studies [49,65,76,77,78]. This finding underscores the patient’s difficulty in effectively controlling biofilm on implants and their structures and the challenge in achieving the elimination of bleeding in all sites in the treatment of PID.

Patients in this study will continue to be monitored and receive PIMT. This follow-up is crucial to assess the long-term maintenance of the clinical and immunological outcomes observed. Given that the treatment effectively reduced pro-inflammatory cytokines involved in osteoclastogenesis, and considering that the surgical therapy protocol used for PI provided adequate access to the implant surface for decontamination, it is expected that crestal bone levels will be sustained and soft tissue inflammation will remain stable over extended follow-up periods, provided that patients stay compliant with the treatment.

This study presents certain limitations, such as the relatively small sample size and short follow-up period, which may restrict the generalizability of the findings. Future research with more extensive sampling and longer follow-up periods would provide more substantial evidence on the modulation of Th17-related cytokine expression and the clinical outcomes of peri-implant disease treatments. Therefore, the results should be interpreted with caution.

## 5. Conclusions

In conclusion, our study demonstrates that after three months of therapy, there was a significant reduction in the expression of cytokines in the peri-implant crevicular fluid of patients with peri-implant mucositis (IL-17A and TNF-α) and peri-implantitis (IL -1β, IL-6, and TNF-α). This finding indicates that peri-implant therapy can modulate important cytokines in exacerbating the inflammatory response and osteoclastogenesis.

## Figures and Tables

**Table 1:** Demographic data at the baseline.

	Total	Peri-Implant Mucositis	Peri-Implantitis	*p* Value
**Participants**	37 (100%)	20 (54.05%)	17 (45.95%)	0.622
**Gender**				
Male	15 (40.54%)	7 (46.66%)	8 (53.34%)	0.457
Female	22 (59.46%)	13 (59.1%)	9 (40.9%)	0.457
**Age**	59.14 (±10.37)	58.65 (±11.62)	59.70 (±9.01)	0.219
**Periodontal Classification in Concomitant Periodontitis**	8 (100%)	2 (25%)	6 (75%)	0.157
Generalized Stage II Grade A	1 (12.5%)	1 (100%)	0 (0%)	-
Generalized Stage III Grade A	6 (75%)	1 (16.67%)	5 (83.33%)	0.197
Generalized Stage III Grade B	1 (12.5%)	0 (0%)	1 (100%)	-
**Last PIMT**				
Less than a year	8 (21.62%)	6 (75%)	2 (2%)	0.157
Between 1 and 2 years	17 (45.95%)	9 (52.94%)	8 (47.06%)	0.808
More than 2 years	12 (32.43%)	5 (41.67%)	7 (58.33%)	0.564

PIMT—Periodontal and peri-implant maintenance therapy. Outcomes were presented as numbers and percentages. Age was presented as mean and standard deviation (±). *p*-value was evaluated using the Chi-square statistical test.

**Table 2:** Clinical outcomes of PIM group in baseline and T1.

	Baseline	T1	*p* Value
PD (mm)	3.90 (±1.34)	3.27 (±1.19)	**0.001**
CAL (mm)	2.41 (±1.37)	1.50 (±1.31)	**<0.001**
% PId	71.43 (±45.58)	46.43 (±50.32)	**0.003**
% BoP	100 (±0.00)	51.79 (±50.42)	**<0.001**

PD—Probing depth; CAL—Clinical attachment level; % PId—percentage of plaque index; % BoP—percentage of bleeding on probing. Outcomes were presented as mean and standard deviation (±). *p*-value was evaluated using the Wilcoxon statistical test.

**Table 3:** Clinical outcomes of PI group in baseline and T1.

	Baseline	T1	*p* Value
PD (mm)	5.29 (±1.74)	3.00 (±1.00)	**<0.001**
CAL (mm)	4.32 (±1.78)	3.00 (±1.80)	**0.004**
% PId	65.63 (±48.25)	46.88 (±50.70)	0.083
% BoP	100 (±0.00)	56.25 (±50.40)	**<0.001**

PD—Probing depth; CAL—Clinical attachment level; % PId—percentage of plaque index; % BoP—percentage of bleeding on probing. Outcomes were presented as mean and standard deviation (±). *p*-value was evaluated using the Wilcoxon statistical test.

**Table 4:** Descriptive data of implants.

		Total Number of Implants (n = 88)	Implants of PIM Group (n = 56)	Implants of PI Group (n = 32)	*p*-Value
Arch, n (%)	Upper	40 (45.45%)	30 (34.09%)	10 (11.36%)	**0.002**
	Lower	48 (54.55%)	26 (29.55%)	22 (25%)	0.564
	*p*-value	0.394	0.593	0.34	
Position, n (%)	Anterior (canine-canine)	13 (14.77%)	8 (14.28%)	5 (15.62%)	0.405
	Posterior	75 (85.23%)	48 (85.72%)	27 (84.38%)	**0.015**
	*p*-value	**<0.001**	**<0.001**	**<0.001**	
Type of prosthetic platform	Morse Taper	40 (45.45%)	32 (57.14%)	8 (25%)	**<0.001**
	External Hexagon	48 (54.55%)	24 (42.86%)	24 (75%)	1.000
	*p*-value	0.394	0.285	**0.005**	
Cemented or screwed	Cemented	40 (45.45%)	27 (48.21%)	13 (40.62%)	**0.027**
	Screwed	48 (54.55%)	29 (51.79%)	19 (59.38%)	0.149
	*p*-value	0.394	0.789	0.289	
Splinted or non-splinted	Splinted	32 (36.37%)	9 (16.08%)	23 (71.88%)	**0.013**
	Non-splinted	56 (63.63%)	47 (83.92%)	9 (28.12%)	**<0.001**
	*p*-value	**0.011**	**<0.001**	**0.013**	

Data presented as number of implants and respective percentage. *p*-value was evaluated using the Chi-square statistical test.

## Data Availability

The original contributions presented in this study are included in the article. Further inquiries can be directed to the corresponding author.
